# AHR Signaling Dampens Inflammatory Signature in Neonatal Skin γδ T Cells

**DOI:** 10.3390/ijms21062249

**Published:** 2020-03-24

**Authors:** Katja Merches, Alfonso Schiavi, Heike Weighardt, Swantje Steinwachs, Nadine Teichweyde, Irmgard Förster, Katrin Hochrath, Beatrix Schumak, Natascia Ventura, Patrick Petzsch, Karl Köhrer, Charlotte Esser

**Affiliations:** 1 IUF—Leibniz Research Institute for Environmental Medicine, Auf’m Hennekamp 50, D-40225 Düsseldorf, Germany; katja.merches@iuf-duesseldorf.de (K.M.); alfonso.schiavi@iuf-duesseldorf.de (A.S.); swantje.steinwachs@iuf-duesseldorf.de (S.S.); nadine.teichweyde@iuf-duesseldorf.de (N.T.); katrin.hochrath@iuf-duesseldorf.de (K.H.); natascia.ventura@iuf-duesseldorf.de (N.V.); 2 Institute of Clinical Chemistry and Laboratory Diagnostic, University of Düsseldorf, Universitätsstrasse 1, D-40225 Düsseldorf, Germany; 3 Immunology and Environment, Life & Medical Sciences (LIMES) Institute, University of Bonn, Carl-Troll-Str. 31, D-53115 Bonn, Germany; heike.weighardt@uni-bonn.de (H.W.); irmgard.foerster@uni-bonn.de (I.F.); 4 Institute of Medical Microbiology, Immunology and Parasitology, University of Bonn, Venusberg Campus 1, D-53127 Bonn, Germany; bschumak@uni-bonn.de; 5 BMFZ—Genomics & Transcriptomics Laboratory, University of Düsseldorf, Universitätsstrasse 1, D-40225 Düsseldorf, Germany; patrick.petzsch@hhu.de (P.P.); koehrer@hhu.de (K.K.)

**Keywords:** γδ T cell, epidermis, aryl hydrocarbon receptor

## Abstract

Background Aryl hydrocarbon receptor (AHR)-deficient mice do not support the expansion of dendritic epidermal T cells (DETC), a resident immune cell population in the murine epidermis, which immigrates from the fetal thymus to the skin around birth. Material and Methods In order to identify the gene expression changes underlying the DETC disappearance in AHR-deficient mice, we analyzed microarray RNA-profiles of DETC, sorted from the skin of two-week-old AHR-deficient mice and their heterozygous littermates. In vitro studies were done for verification, and IL-10, AHR repressor (AHRR), and c-Kit deficient mice analyzed for DETC frequency. Results We identified 434 annotated differentially expressed genes. Gene set enrichment analysis demonstrated that the expression of genes related to proliferation, ion homeostasis and morphology differed between the two mouse genotypes. Importantly, with 1767 pathways the cluster-group “inflammation” contained the majority of AHR-dependently regulated pathways. The most abundant cluster of differentially expressed genes was “inflammation.” DETC of AHR-deficient mice were inflammatory active and had altered calcium and F-actin levels. Extending the study to the AHRR, an enigmatic modulator of AHR-activity, we found approximately 50% less DETC in AHRR-deficient mice than in wild-type-littermates. Conclusion AHR-signaling in DETC dampens their inflammatory default potential and supports their homeostasis in the skin.

## 1. Introduction

The skin is in continuous contact with the environment and protects the body from dehydration, chemicals, and pathogens. A network of different resident immune cell types in dermis and epidermis critically influences the homeostasis of skin cells and ensures skin integrity. In mouse and man, the skin harbors γδ T cells, which are critical for fighting pathogens, promoting wound healing and deleting cancer cells (reviewed in [1,2]). The resident epidermal lymphocyte population in mice almost completely consists of γδ T cells, while in humans αβ T cells are more frequent [3,4]. Murine epidermal γδ T cells carry an invariant Vγ3Vδ1-T cell receptor (Garman’s nomenclature [5]) and are also called dendritic epidermal T cells (DETC) because of their unique morphology. Their genetic ablation results in increased susceptibility to chemical-induced skin tumors and reduced inflammatory responses to stress [6,7]. DETC develop in the fetal thymus during a small time-window [8] and then migrate to the epidermis, where they undergo a short period of extensive proliferation with a peak around two weeks after birth [9]. Slow self-renewal and continuous moderate T cells receptor (TCR)-activation at synapses formed at the junctions between keratinocytes ensure their maintenance [10,11,12]. We previously showed that DETC from aryl hydrocarbon receptor (AHR)-deficient mice (*Ahr* ^-/-^) failed to express the receptor tyrosine kinase C-Kit (KIT) because *Kit* is a direct target gene of the AHR [4,13]. We, therefore, suggested that KIT, similarly to the situation in gut intraepithelial lymphocytes [13], is important for the maintenance of DETC. The AHR is a ligand-activated transcription factor, originally known for controlling genes coding for xenobiotic metabolism enzymes. AHR´s function has also been linked to cytokine secretion, immune cell differentiation, skin barrier strength, and many others (reviewed in [14,15]). AHR signaling typically induces and suppresses a set of functionally related genes (“batteries”) depending on cell type and context [16,17,18]; it is thus likely, that beyond *Kit,* other genes are modulated by AHR and relevant for establishment and function of DETC. In addition to the lack of proliferation, DETC in AHR-deficient mice display a rounded morphology with less extensive dendrites [4]. A similar morphologic switch is known for DETC in wounded skin [19,20] and has been interpreted as a possible sign of activation [21,22]. 

DETC can be activated via stress molecules from damaged or transformed/cancerous keratinocytes. For instance, retinoic acid early transcript 1 (Rae-1) triggers the natural killer (NK) receptor NKG2D present on all DETC [6,23]. Depending on the route of activation, this results in potent cytotoxicity against cancer cells [24] or fast secretion of cytokines such as interferon-γ (IFNγ) and other mediators of inflammation. As sentinels of skin health, DETC need to balance skin inflammation, i.e., discern “danger” and restrain their inflammatory potential. DETC receive signals continuously via their TCR, yet these signals do not trigger acute inflammatory activity, and may just serve to ensure self-renewal of the DETC by proliferation [11,19]. We previously proposed that a balanced AHR-activation is important for skin homeostasis in a healthy and inflamed situation [15]. 

We here analyze the gene expression profile of sorted DETC at the time point of the proliferation burst and identify gene clusters relevant for both inflammation and morphology as major targets of AHR-signaling at this time point. Our data point to a critical function of AHR in controlling the homeostasis and inflammatory potential of DETC. 

## 2. Results

### 2.1. Establishment of DETC in the Epidermis Depends on AHR and AHRR, But Not on KIT

**Figure 1:**
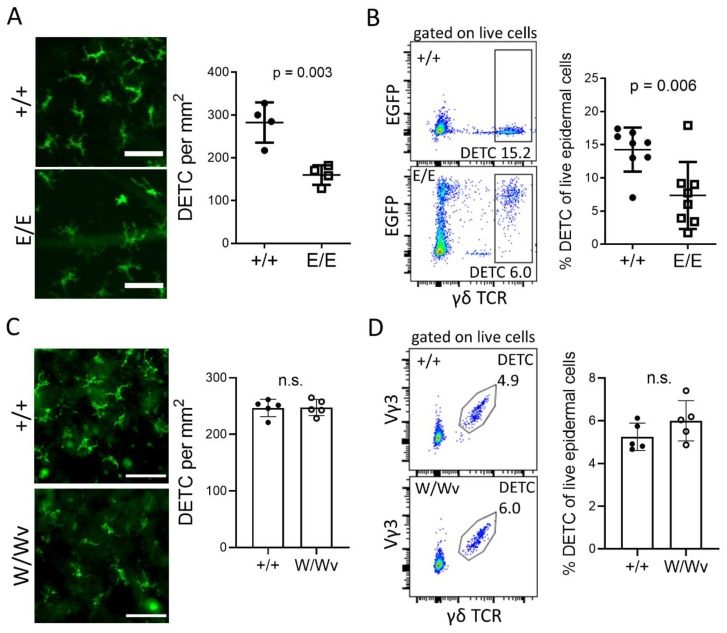
The establishment of dendritic epidermal T cells (DETC) depends on aryl hydrocarbon receptor (AHRR) response, but not on KIT. (**A**) Representative pictures of epidermal earsheets from 10–12 weeks old Ahrr ^+/+^ and Ahrr ^E/E^ mice stained with anti-Vγ3-FITC (green) and quantification of DETC on the sheets (scale bar = 50 µm, n = 4 mice) (**B**) FACS-analysis of DETC, stained by γδTCR-APC in overnight cultivated epidermal cell suspensions of 10–12 weeks and 8–12 months old Ahrr ^+/+^ and Ahrr ^E/E^ mice (20000 events are displayed; 2 experiments, n = 7–8 mice) (**C**) Representative pictures of epidermal sheets of 10–13 months old Kit ^+/+^ or Kit ^W/Wv^ mice stained with anti-Vγ3-FITC (green) and quantification of DETC on the sheets (scale bar = 50 µm, n = 5 mice) (**D**) FACS-analysis of DETC, stained by γδTCR-APC and Vγ3TCR-FITC in overnight cultivated epidermal cell suspensions of 10–12 weeks old Kit ^+/+^ or Kit ^W/Wv^ mice (20,000 events are displayed; 1 experiment, n = 5 mice) (**A–D**) Statistics were calculated with parametric *t*-tests.

We had previously shown that the DETC in *Ahr ^-/-^* mice migrated into the epidermis upon their generation in the thymus, but proliferated less than *Ahr* ^+/+^ DETC and that expression of the AHR target gene *Kit* was lower compared to wild-type *Ahr* ^+/+^ littermate mice. As KIT regulates the activation and expansion of γδ T cells in the gut [25], we had suggested that AHR-mediated targeting of KIT expression underlies the low proliferation of DETC in the skin as well. The AHR-repressor (AHRR) is encoded by an AHR target gene and believed to repress AHR-dependent gene transcription [26]. We hypothesized that AHRR-deficient mice have higher expression of *Kit* and therefore more DETC in their epidermis. Therefore, we analyzed mice, in which the *Ahrr*-gene was replaced by a green fluorescent protein reporter construct (EGFP) [27]. The frequency of DETC in AHRR-deficient mice (*Ahrr* ^E/E^) was reduced by half compared to *Ahrr* ^+/+^ mice (Figure 1A,B). Interestingly, sorted DETC from these mice did not show a reduction but rather a slight enhancement of *Kit* expression (Supplementary Figure S1A). This suggested that the establishment of DETC does not necessarily depend on KIT. Indeed, mice with a *Kit* ^W/Wv^ mutation, which leads to a reduced KIT-activity and loss of KIT-dependent cells like mast cells, melanocytes, intraepithelial lymphocytes, and intestinal lymphoid cells [13,25,28,29], had a normal frequency of DETC in their epidermis (Figure 1C,D). We conclude that both AHR- and AHRR-signaling is important for DETC homeostasis, independently of KIT, and turn again to our AHR-deficient mouse model to characterize AHR-dependent activity in DETC more closely.

### 2.2. DETC of Ahr ^-/-^ Mice Express More Inflammatory and Less Actin-Modulating Genes

**Figure 2:**
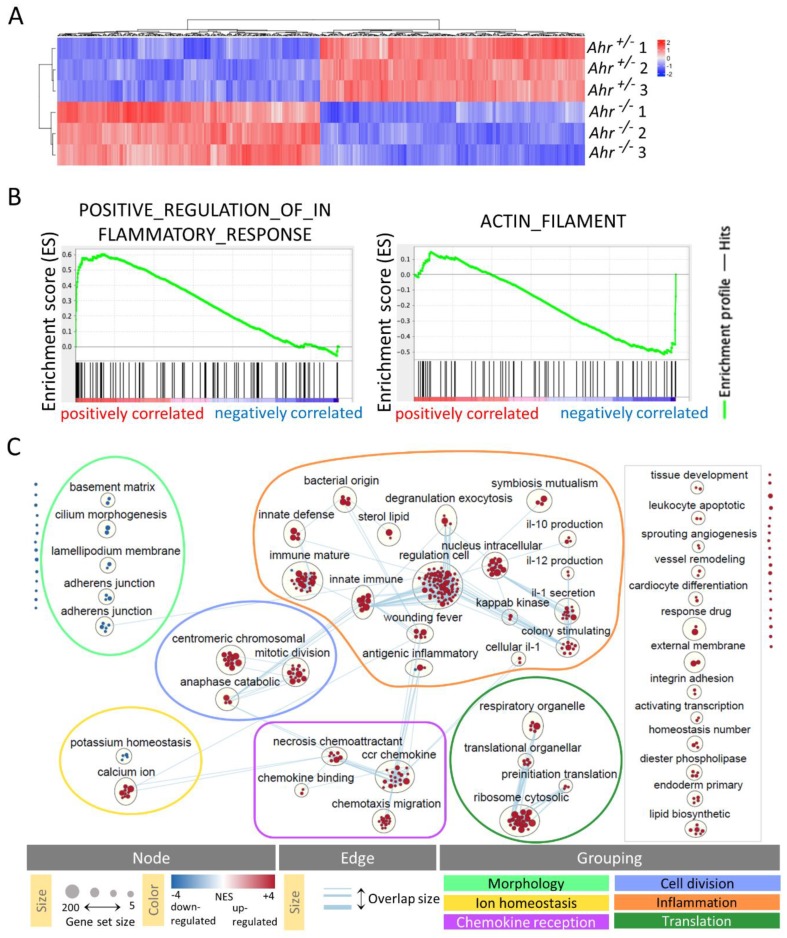
*Comparative analysis of the transcriptome of Ahr ^-/-^ and Ahr ^+/-^ dendritic epidermal T cells (DETC).* RNA of DETC, isolated by fluorescent activated cell sorting (FACS) from 2–3 mice two weeks of age, was pooled for one sample and analyzed on a microarray. Thereby data from 3 arrays for each *Ahr ^+/-^ and Ahr ^-/-^* DETC were generated (n = 3 arrays). (**A**) Heatmapped expression levels of genes differentially regulated with an FDR adj. *p*-value (*q*-value) *q* < 0.5 (**B**) selected enrichment plots for one positive associated pathway (left panel; *p*-value < 0.001; *q*-value = 0.004) and one negative associated pathway (right panel; *p*-value = 0.004; *q*-value = 0.555). (**C**) Overall thumbnail view of the publication-ready enrichment map created with parameters nominal *p*-value < 0.01, which includes pathways up to a *q*-value < 0.6, and edge-cut-off 0.4. Red and blue nodes represent pathways positive- or negative-associated with *Ahr*-deletion, respectively. Nodes were manually laid out to form a clearer picture. Clusters of nodes were labeled using the AutoAnnotate Cytoscape application and were subsequently renamed by removing of one of three words for more clarity. Individual node labels were removed for clarity using the publication-ready button in EnrichmentMap. The grouping of node-clusters was performed manually under consideration of the similarity of biological processes indicated by the wording of the clusters and contained pathways and edge-connections (gene-overlaps) between clusters. Clusters surrounded by the grey rectangle were not connected to other clusters or each other and were not considered in further analysis. A legend was manually added at the bottom of the figure.

To identify genes, which might affect the DETC-proliferation in an AHR-deficient situation, we performed RNA-microarray analysis of DETC sorted to high purity from *Ahr* ^+/-^ or *Ahr* ^-/-^ mice at two weeks of age, the peak time of the so-called “proliferation burst” of DETC in wild-type mice [9]. The proportion of DETC among live epidermal cells was between 10%–15% in *Ahr* ^+/-^, similar to *Ahr* ^+/+^ mice [4], and 2%–4% in *Ahr*^-/-^ mice. The gene expression profiles of *Ahr* ^+/-^ versus *Ahr* ^-/-^ DETC revealed 434 annotated genes at a false discovery rate (FDR) adjusted *p*-value ≤ 0.05 (Figure 2A). Approximately half of the genes were up- (218) and down-regulated (216) in *Ahr ^-/-^* versus *Ahr ^+/-^*. As expected, the AHR-target genes *Kit* (FC = −1.22, FDR adj-*p* = 0.006) and *Ahrr* (FC = −1.44, FDR adj.-*p* = 0.003) were down-regulated.

We used gene-set enrichment analysis (GSEA; http://software.broadinstitute.org) to determine the gene ontology (GO) pathways that were positively or negatively correlated to the *Ahr ^-/-^* genotype. For example, genes of the positively associated pathway “positive regulation of inflammatory response” were to a large part up-regulated and genes of the negatively associated pathway “actin filament” tended to be down-regulated in *Ahr* ^-/-^ DETC (Figure 2B). In total, GSEA revealed 144 pathways, which positively correlated with the *Ahr* ^-/-^ -genotype at *p*-values of *p* < 0.01 and FDR-adjusted *p*-values (q-values) of *q* < 0.05 and 34 pathways, which negatively correlated at *p*-values *p* < 0.01 and *q*-values *q* < 0.6. Using the EnrichmentMap-App in Cytoscape these pathways were clustered by the Cytoscape-App AutoAnnotate (Figure 2C) according to the protocol of Reimand et al. [30]. By further manual comparison of the biological processes behind the pathways and the overlaps between the different clusters we identified six cluster-groups and named them according to their most obvious function. We tagged “inflammation,” “chemokine reception,” “translation,” “cell division” from the positively correlated pathway-clusters. The group “ion homeostasis” contained positively and negatively-associated pathway-clusters and the rest of negative-associated pathway-clusters could be combined in the group “morphology”’ (Figure 2C). With 1767 pathways the cluster-group “inflammation” contained the majority of AHR-dependently regulated pathways, indicating a leading role of AHR-signaling in the transcription profile for this function. Note that in the absence of AHR the genes in these pathways were in most cases up-regulated, suggesting that AHR dampens their expression in wild-type DETC. 

### 2.3. DETC of Ahr ^-/-^ Mice are Inflammatory Active and Have Altered Calcium and F-Actin Levels

We verified several genes, which we considered representative for each of the six identified cluster-groups by quantitative PCR. For the group “inflammation,” we chose characteristic and functionally described inflammatory factors produced by DETC, which were differentially regulated, such as IFNγ, granzyme F (GZMF), or programmed death-ligand 1 (PDL-1) [31]. For the other groups, we assessed those genes, which most frequently appeared under the top three leading-edge-genes in all pathways of one cluster-group [32]. 

**Figure 3:**
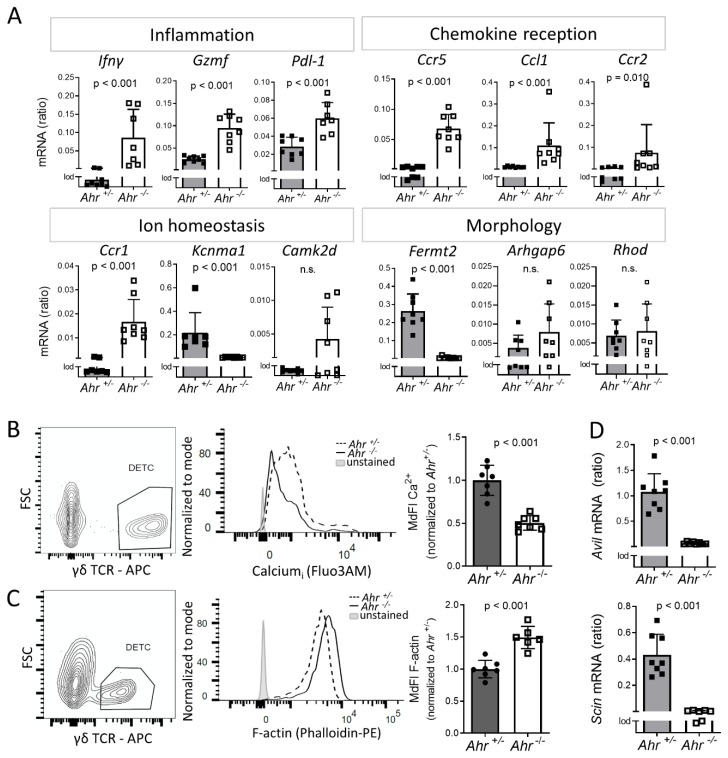
*Verification of gene-expression and effects of Ahr-deletion in DETC on calcium and F-actin*. (**A**,**D**) RNA of DETC, FACS-sorted from 2 weeks old mice was isolated. Expression of indicated genes was measured by qPCR; shown are ratios to the housekeeper gene *Rps6* (*n* = 8 mice; *t*-test for *Gzmf*, *Pdl1*, *Ccr5*, *Rhod, Avil, Scin*; Mann–Whitney-test for remaining genes). (**B**) epidermal cells were isolated from 4–8 weeks old mice, stained with Fluo3AM and antibodies against γδTCR and analyzed by flow cytometry; shown are the median fluorescent intensities of Fluo3AM (MdFI) normalized to *Ahr ^+/-^* mice. Dead cells were excluded by DAPI-staining (3 experiments, *n* = 7 mice). (**C**) epidermal cells were isolated from 8–11 weeks old mice, stained with phalloidin-PE and antibodies against γδTCR and analyzed by flow cytometry; shown are the median fluorescent intensities of phalloidin (MdFI) normalized to *Ahr ^+/-^* mice (2 experiments, *n* = 6–7 mice (**B**,**C**) statistical test: Mann–Whitney-test.

As GSEA analyzes the gene-expression data without any threshold for significance, also weakly regulated genes with poor statistical power are included. The selected leading-edge genes in the cluster-group “cell division” and “translation” showed only weak or non-significant changes, which was in line with the microarray data (Supplementary Figure S1E). The genes of the cluster-groups “inflammation” and “chemokine reception” were strongly up-regulated (Figure 3A). Functionally, IFNγ is an immunostimulatory cytokine and GZMF serves as a mediator of cytotoxicity when transferred into the target cell [33]. PDL-1 can be induced by IFNγ and regulates T-cell-responses [34,35,36]. The higher gene-expression activity of inflammatory genes was congruent with a measurement of intracellular IFNγ and GM-CSF in DETC of older *Ahr ^+/+^* and *Ahr ^-/-^* mice, which showed a low but still higher frequency of IFNγ+ and GM-CSF+ DETC in *Ahr ^-/-^* mice compared to *Ahr ^+/+^* mice (Supplementary Figure S2). CCL1 attracts regulatory T cells by binding to CCR8 [37]. CCR2 and CCR5 can guide γδ T cells to sites of inflammation [38]. Together with CCR1, they bind several chemokines including CCL3 and CCL5 and can mobilize calcium after ligand-binding [39]. Expression of *Kcnma1*, which codes for a subunit of calcium-regulated large conductance (BK) potassium channels [40], was almost blunted in *Ahr ^-/-^* cells, while calcium/calmodulin-dependent protein kinase II delta (*Camk2d*) showed no significant differential regulation and was in both genotypes close to the detection limit (Figure 3A). In contrast to the microarray data, Rho GTPase-activating protein 6 (*Arhgap6*) and the Rho GTPase (*Rhod*) did not differ in their RNA content between *Ahr ^+/-^* and *Ahr ^-/-^* DETC; they were almost undetectable. Expression of fermitin family member 2 (*Fermt2*), whose product is also called Kindlin-2, and is known to link actin-structures to podocyte morphology [41], was strongly down-regulated in *Ahr ^-/-^* DETC. In T cells the related protein Kindlin-3 has been shown to coordinate F-actin, which enables integrin-signaling and migration [42]. Whether or not AHR-mediated FERMT2 expression relates to the DETC morphology is not clear.

Given these gene expression patterns, we further speculated that levels of intracellular calcium would be affected in DETC of adult *Ahr ^-/-^* mice. Indeed, the intracellular calcium-levels measured by flow-cytometry with Fluo3am were significantly reduced in *Ahr ^-/-^* DETC compared to *Ahr ^+/-^* controls (Figure 3B). A possible consequence of the impaired expression of *Fermt2* is a change in the amount of filamented actin (F-actin). Flow-cytometry measurement using fluorescently labeled phalloidin showed that DETC isolated from adult *Ahr ^-/-^* mice had elevated intracellular F-actin-levels (Figure 3C). In line with this, other actin-modulating genes like *Advillin* and the known AHR-target gene *Scinderin* [43], both members of the actin-severing gelsolin-superfamily [44,45,46] were shut down in DETC of *Ahr ^-/-^* mice (Figure 3D; GEO accession: GSE142437). 

To determine whether these effects were due to intrinsic AHR-deletion or might be induced by surrounding AHR-deficient cells. *Ahr*-flox-K5-cre x *Ahr*-flox-LC-cre mice carrying an *Ahr*-deletion only in keratinocytes and Langerhans cells had normal amounts of DETC (Supplementary Figure S1C). Furthermore, DETC of *Ahr*-flox-K5-cre mice showed no similarities to DETC of *Ahr ^-/-^* mice in the up-regulation of *Ifnγ* and *Gzmf* or down-regulation of *Scinderin* and *Kcnma1* (Supplementary Figure S1D). Finally, ablating AHR in γδT cells of adult mice using a mouse line with a tamoxifen-inducible *Cre* expression in the delta-gene, led to a slow but eventually significant loss of DETC in the skin after 4–8 weeks (Supplementary Figure S1E). Notably, when the AHR-deletion was induced in newborns the loss of DETC occurred already after 15 days (Supplementary Figure S1F). Together, this indicates that the loss of proliferative capacity and probably also the dramatic gene expression changes in DETC are likely triggered by intrinsic *Ahr*-deletion. 

Together, these data verify the findings in the microarray-data and indicate (i) that AHR-deficiency increases the inflammatory tone of DETC, and (ii) that genes responsible for actin-metabolism and ion-homeostasis need AHR-signaling. 

We next used the genome search published by Sun et al. [47] to identify putative AHR-responsive elements, the so-called xenobiotic response elements (XRE) in the promoters of the leading-edge genes from all negatively correlated (*p* < 0.01) pathways. Of these selected genes (genes redundant in different pathways were only counted once), 122 of 285 had putative XRE-sequences in their promotors and 66 of those were significantly (*p* < 0.05) down-regulated in DETC of *Ahr ^-/-^* mice, among them *Fermt2* and *Advillin* (Table 1). 

Genes with at least 1 XRE in their promotors were identified from leading-edge genes of all negatively associated pathways (Figure 2C, “morphology”). Shown are genes expressed significantly lower in *Ahr ^-/-^* DETC according to the nominal *p*-value < 0.05. Genes in the table are sorted according to the number of pathways, in which they appear as leading-edge-genes (relevance). Where this number is the same, they are sorted in order of the *p*-value. Genes down-regulated at an adjusted *p*-value < 0.05 are highlighted in bold letters.

### 2.4. Lack of IL-10-Signaling Does not Affect DETC Homeostasis

**Figure 4:**
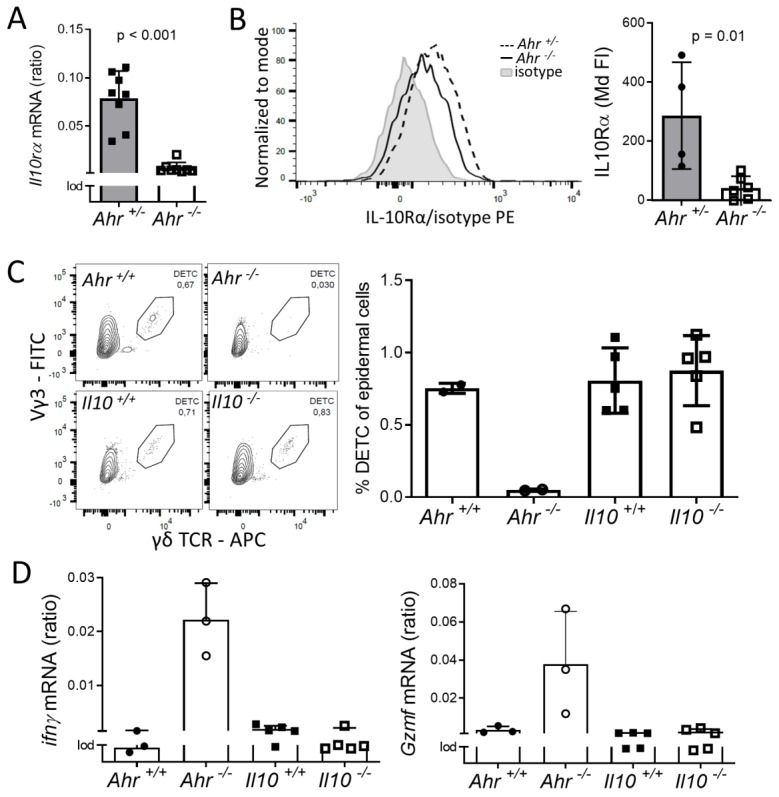
*The influence of IL-10 signaling on DETC maintenance.* (**A**) RNA of DETC, FACS-sorted from 2 weeks old mice, was isolated. Expression of *Il10ra* was measured by qPCR; shown are ratios referred to the housekeeper gene *Rps6* (*n* = 8 mice; Mann–Whitney-test). (**B**) isolated epidermal cells from 4–10 weeks old *Ahr ^+/-^* or *Ahr ^-/-^* mice were stained with antibodies against γδTCR and IL10Rα or and isotype-antibody and analyzed by flow cytometry. Median fluorescent intensities (MdFI) of isotype-controls were subtracted from the MdFI of IL10Rα and normalized to *Ahr* ^+/-^ DETC (3 experiments, *n* = 4–6 mice, Mann–Whitney-test) (**C**) overnight cultivated epidermal cells from 6–8 weeks old mice were stained with antibodies against γδTCR and Vγ3 and analyzed by flow cytometry (50000 events are displayed; *n* = 2–5 mice) (**D**) RNA was isolated from DETC FACS-sorted from 9–20 weeks old *Il-10 ^+/+^* or *Il-10 ^-/-^* mice; expression of *interferon-γ* (*Ifnγ*) and *granzyme-F* (*Gzmf*) was measured by qPCR; shown are ratios referred to the housekeeper gene *Rps6* (*n* = 3–6 mice).

The inflammatory activity of T cells is regulated by several mechanisms including signals from inhibitory auto- or paracrine acting cytokines. We hypothesized that the down-regulation of receptors for such cytokines might have led to the increased inflammatory signature in DETCs of *Ahr ^-/-^* mice. One potent regulator of T cell activity is IL-10, which binds to IL-10-receptor (IL-10R) [48]. The microarray results revealed that *Il10rα* expression was decreased in DETC of *Ahr ^-/-^* mice (fold change −2.27, adj. *p*-value < 0.001, Figure 4A). IL-10Rα-protein on the surface of *Ahr ^-/-^* DETC was significantly and strongly reduced by approximately 90% (Figure 4B). *Il10rβ* was not affected (GEO accession: GSE142437). Human IL-10 inhibits the secretion of IFNγ by αβ and γδ T cells [49]. The impact of IL-10 on murine DETC is unknown. Therefore, we addressed the question of whether the loss of IL-10 signaling through the IL-10R might push the DETC into an inflammatory condition and prevent them from proliferation and forming dendrites. To answer this, we analyzed DETC from *Il-10 ^-/-^* mice. DETC from *Il-10 ^-/-^* mice were normal in number—we found a cell density of 318 DETC/mm2 (SD 13.7; *n* = 3 mice) or about 1% of epidermal cells (Figure 4C), and the cell morphology was normal (Supplementary Figure S1B). Furthermore, we could merely detect expression of the inflammatory cytokines *Ifnγ* or *Gzmf* (Figure 4D). Therefore, these data indicate that lack of IL-10 signaling does not impact the establishment and inflammatory condition of DETC in a non-inflammatory situation.

## 3. Discussion

Skin-resident DETC are a vital part of the murine skin innate immune defense. We had previously shown that AHR-signaling is necessary for their establishment in the epidermis. In AHR-deficient mice, DETC fail to proliferate during the critical “proliferation burst”-phase in the first 2–4 weeks after birth [4], and display fewer of the characteristic dendrites. The AHR is a transcription factor with the capacity to target many genes. Assuming that gene expression changes underlie the fate of the DETC, we assessed the gene expression profile two weeks after birth at the height of the proliferation phase. 

In DETC of *Ahr ^-/-^* mice, the expression of 434 genes differed compared to DETC from their *Ahr ^+/-^* littermates. Half of these genes were down-regulated, indicating that AHR controls their expression, either directly or indirectly. The AHR is considered a transcriptional activator. However, studies showed that AHR activity may decrease gene expression as well [17,47,50]. Mechanisms like transcriptional induction of upstream suppressors of a given gene, interaction with co-repressors in the transcriptional complex itself or steric inhibition of other transcription factors at the promoters have been suggested [51]. This points to the importance of the modular promoter set-up as a basis for cell-specific usage of the AHR as we have suggested before [52]. For instance, the group of Kaminski recently demonstrated that binding of AHR to the early B cell factor 1 (EBF1) promoter results in the down-regulated expression of this B cell-specific differentiation factor [53]. In our present study, we identified 144 genes, which were up-regulated in DETC of *Ahr ^-/-^* mice, i.e., AHR-presence would impair their expression. We hypothesized that the failure of DETC to proliferate and to maintain themselves in the skin would be reflected by the regulation of genes related to proliferation and cell morphology and genes relevant for the forming of contact zones with the surrounding cells. Indeed, pathways related to cell morphology and ion homeostasis were negatively associated with AHR-deletion. Likewise, the expression of genes coding for F-actin modulating enzymes like *Fermt2, Scinderin* and *Advillin* and ion channels like *Kcnma1, Kcng3* and *Kcnab* were down-regulated in DETC of *Ahr^-/-^* mice. These gene expression changes were correlated with increased F-actin and decreased intracellular calcium levels in the cells. 

It remains to be investigated, if some of those genes are targeted by the AHR directly and if the AHR is thereby able to impact on cytoskeleton and ion homeostasis. In αβ T cells the directed actin-polymerization in the nucleus and cytoplasm is triggered and controlled by the intracellular calcium concentration [54,55]. Despite the difference in the structure between the αβTCR and γδTCR and the lack of CD3δ subunit in the murine γδTCR-complex, the triggered signaling cascades include linker of activated T cell (LAT)-phosphorylation and calcium mobilization in both cell types [56,57]. Thus, the regulation of the turnover of actin-filaments in combination with aberrant intracellular calcium concentration are factors, which might influence DETC morphology and down-stream proliferation. DETC need to reassemble their dendritic morphology for cell division [19]. It is also of interest in this context that actin filament organization regulates cell effector functions. This is evident by treatment with cytochalasin, which inhibits actin polymerization and leads to insufficient T cell activation [58]. In non-immune cells, adenocarcinoma cells or lens epithelial cells, a pharmacological modification of actin-filament polymerization was sufficient to induce ion channel activity [59,60]. It would be interesting to assess if modifications of actin-filament assembly are sufficient to influence the activity and proliferation of DETC. Our findings regarding actin-filaments could also be meaningful for other dendrite-forming cells like neurons, and the role of AHR in such cells. This warrants further investigation. Finally, the morphological changes in DETC of neonatal AHR-deficient mice, and the gene changes in the pathway “morphology” may reflect on the role of AHR regarding an impaired transport in the dendrites themselves. Steady-state DETC constantly internalize their TCR-molecules, thereby establishing a dynamic “polarized conduit system,” which might serve in probing their environment, and internalizing or secreting proteins [61]. 

While up to 14 XREs were present in some promoters of down-regulated genes, we asked whether the number of XREs would correspond to the expression level. However, we did not find a correlation between the number of XREs and fold-change differences. For instance, *Kit* has 14 XREs in its promoter, but the fold-change in expression in AHR-deficient versus -proficient DETC was lower compared to Interleukin 6 signal transducer (*IL6st*) with only 1 XRE. Overall, AHR-activation is driven by many factors and is highly context-dependent, including promoter/enhancer structure and spatial distribution [15,18]. Some of the genes, which differed between DETC of *Ahr ^-/-^* and *Ahr ^+/-^* mice, did not even have XREs. Conceivably, this reflects a cascade-like gene regulation or secondary gene modulation effects for these genes.

A surprising and most interesting finding of the gene expression analysis was the dominance of inflammation-related genes, which were up-regulated in the DETC of *Ahr* ^-/-^ mice. In line with this, the up-regulation of *Ifnγ* in conventional T cells with AHR-deletion has been reported [17,62]. The “inflammatory gene expression profile” hints at a general role of AHR in dampening inflammatory DETC activity, which might be harmful to the skin, if it remains uncontrolled. Interestingly, the intracellular staining for IFNγ and GM-CSF-protein showed that only some DETC in 8 weeks old *Ahr ^-/-^* mice were positive for IFNγ or GM-CSF. This might be due to a local activation signal. DETC likely recognize molecules from stressed or dying keratinocytes or aberrant surface molecules from transformed cells. It is reasonable to assume that there must be some kind of threshold for activation, as stressed keratinocytes will exist also in healthy skin, and we suggest that AHR might be an important factor in such a balance. Similar thoughts were proposed for Langerhans cells, another potent immune cell type in the epidermis [63]. In agreement with this idea, not only were inflammatory genes expressed more strongly in DETC of *Ahr ^-/-^* mice, but also anti-inflammation modulators somewhat less, such as *Il10rα, Il6st,* and *Il12rb1*. Of note, we could not detect more TCR-stimulation in DETC of *Ahr* ^-/-^ mice, as the expression of *Nur77*, as a marker for TCR-stimulation [64], was even lower in *Ahr* ^-/-^ DETC (GEO accession: GSE142437), which indicates that the DETC were activated over an alternative mechanism e.g., through NKG2D. The exacerbated inflammatory phenotype may be a harbinger of the loss of DETC, which will occur within the next weeks of the mouse’s life. 

Is DETC-intrinsic AHR-activity responsible for the effects? Our data show that DETC from mice, in which the AHR was conditionally deleted in the most abundant DETC-surrounding cells, keratinocytes or Langerhans cells, developed normally, which strongly suggests an intrinsic effect. Furthermore, the ablation of AHR in DETC via tamoxifen in older mice led to a slow (and in newborn mice to a faster) disappearance of DETC, strongly supporting the notion of an intrinsic effect on AHR-dependent DETC-proliferation. However, we did not yet analyze DETC-function during DETC-development after tamoxifen-induced intrinsic AHR-ablation. As tamoxifen interacts with the estrogen-receptor, which might interfere in DETC-function and AHR-signaling, this mouse model has to be analyzed in more detail to reveal DETC-intrinsic AHR-dependent mechanisms. The fact that only few DETC produced IFNγ or GM-CSF in 8 weeks old *Ahr ^-/-^* mice indicates that also in younger mice the inflammatory phenotype requires additional local signals, which might be facilitated by extrinsic factors such as the impaired skin barrier in *Ahr ^-/-^* mice [16]. DETC-proliferation depends on IL-2, IL-15, and IL-7 produced by keratinocytes [65]. Neither of these cytokines or their receptors was expressed significantly lower in the DETC (GEO accession: GSE142437) or the total epidermis [4], so it is fair to state that DETC in *Ahr ^-/-^* mice did not suffer from growth-factor deprivation. Our previous microarray analysis of the whole epidermis revealed that the DETC survival factor SKINT1 in keratinocytes is also not down-regulated upon AHR-deletion (GEO accession: GSE80273). Of note, the recently identified T-cell-survival factor TOX [66] was not differentially expressed (GEO accession: GSE142437). Interestingly, our data show that DETC can express the programmed death-ligand 1 *Pdl1 (CD274,* formerly *B7-H1)*, which to our knowledge was not reported before. By the expression of PDL-1 DETC might be able to regulate local T cell responses and thereby modulate skin inflammation.

We assessed two possible “extrinsic” factors in vivo, which might drive DETC loss, namely IL-10 and the tyrosinase and stem cell factor receptor KIT. The receptor for the immunosuppressive cytokine, IL-10Rα, was significantly down-regulated in DETC of *Ahr ^-/-^* mice. *Il10rα*-up-regulation in thymocytes by the high-affinity AHR ligand 2,3,7,8-tetrachlorodibenzo-*p*-dioxin (via an upstream transcription regulator KLF2) has been reported more than a decade ago [67] and *Il10rα* was identified as an AHR-target gene in intestinal epithelial cells [68]. Surprisingly, DETC numbers were normal in the epidermis of *Il10*-deficient mice, thus, the lower *Il10rα*-expression likely was of no consequence. This might be different in a stressful situation. In the epidermis, IL-10 is up-regulated in response to mechanical stress [7] and IL-10 deficiency augments skin inflammation [69]. Even more surprising was our finding that the DETC numbers were normal in *Kit* mutant mice. Mice with a *Kit* ^W/Wv^ mutation have a reduced KIT-activity and lose KIT-dependent cells like mast cells, melanocytes, intraepithelial lymphocytes and intestinal lymphoid cells [13,25,28,29]. We had previously demonstrated that KIT expression is low in DETC of *Ahr ^-^*^/-^ mice, and that *Kit* is a direct target of the AHR with several functional XREs in its promoter [4]. We had therefore suggested that AHR-dependent *Kit* expression is potentially involved in DETC homeostasis. In contrast to this expectation, we here demonstrate that *Kit* ^W/Wv^ mice displayed a normal frequency and morphology of DETC in their epidermis. Congruent with a role for AHR signaling in DETC homeostasis, however, was the finding that in AHR repressor-deficient mice, DETC did not establish. AHRR´s functions are complex. In colonic intraepithelial lymphocytes, AHRR prevents excessive IL-1β production, a function underlining again the importance of the AHR signaling pathway in inflammation modulation [27]. Nonetheless, the mechanisms for DETC-loss at AHRR deficiency remain to be investigated. 

Not only Vγ3 T cells need the AHR for their residence in the epidermis: It has been shown that the presence of intact AHR-signaling is an advantage for regulatory memory T cells (T_RM_) to reside in the epidermal niche. Transferred *Ahr* ^+/+^, but not *Ahr* ^-/-^ skin-resident memory αβ T cells, which migrate into the epidermis in similar numbers upon infection or irritation, stably establish over time [70]. Furthermore, αβ T cells do not replace the lost DETC in *Ahr ^-/-^* mice [4]. This underscores that the AHR acts to facilitate the cell-establishment in the epidermal niche independently of TCR-specific stimulation.

In conclusion, we provide evidence that DETC require intact AHR signaling for expression of genes relevant to DETC establishment and maintenance in the epidermis in the critical phase after birth. In addition, our data strongly suggest a dampening role for AHR on the inflammatory profile of DETC in healthy skin. 

## 4. Materials and Methods 

### 4.1. Animals 

Female and male *Ahr ^-/-^* mice, in which the exon 2 of the *Ahr* was deleted [71] and *Ahrr ^EGFP^* mice (Ahrr ^E/E^ and Ahrr ^+/+^), in which the *Ahrr*-gene was replaced by a green fluorescent protein reporter construct (EGFP) [27] were bred under SPF-conditions in our animal facility. Wild-type or heterozygous littermates were used as control animals [4]. *Ahr ^fl/fl^* x Langerin-Cre and *Ahr ^fl/fl^* x K5-Cre mice [4,72] were also housed in our facility and cross-bred for the generation of *Ahr ^fl/fl^* K5-Cre Langerin-Cre mice, in which the exon 2 of the *Ahr* is deleted only in Langerhans cells and keratinocytes, respectively. TCRdCreERS-RFP-*Ahr*flox mice were crossed from a tamoxifen-inducible *Cre*-TCRd line [10] with a ROSA26-RFP line [73], which were a kind gift from Immo Prinz at the Hannover Medical School, and then crossed with *Ahr*-floxed mice. The resulting line *Ahr*-flox/iCRE RFP deletes *Ahr* specifically in γδ T cells upon tamoxifen injection with the highest efficiency in DETC. The deletion can be followed easily as the fluorescent protein RFP becomes simultaneously expressed in the *Cre*-expressing DETC. Adult mice were injected intraperitoneally with 1 mg tamoxifen (Sigma Aldrich, St. Louis, MO, USA) in 100 µL corn oil (Sigma Aldrich) 3 times every other day. Neonatal mice were fed beginning at the age of 1 day for 5 days daily with 5 drops of corn oil containing 10 mg/mL tamoxifen. Skin tissue from IL-10 deficient mice on the C57BL/6 background and C57BL/6 wild-type controls was prepared from 6–20 week old female and male donor mice. *Il-10 ^-/-^* mice and co-housed wild-type control mice were bred at the House of Experimental Therapy (HET) of the University of Bonn [74] and in the animal facility of Hannover Medical School [75] under SPF conditions. Female 10–13 months old WBB6F1 *Kit ^W/Wv^* and *Kit ^+/+^* littermates were kindly provided by Hans-Reimer Rodewald, DKFZ, Heidelberg. Water and food were provided ad libitum. All animal experiments were done with the permission of the relevant German government agency (Landesamt für Natur, Umwelt und Verbraucherschutz, registration number 84–02.04.2015-A566, from 24 May 2016).

### 4.2. Immunohistochemistry of Epidermal Ear Sheets

Mice were sacrificed and ear skin sheets prepared. Skin was placed dermal sides down in 20 mM EDTA in 1X phosphate-buffered saline (PBS) for 3.5 h at 37 °C. Subsequently, the epidermis was peeled off and placed on a glass slide for staining. Unspecific binding sites were blocked with 10% fetal calf serum (FCS, PAN-Biotech, Aidenbach, Germany) in PBS for 1 h at room temperature. DETC were stained with anti-Vγ3-TCR-FITC (clone 536; #553229 BD Biosciences, San Jose, CA, USA) diluted to 2.5 µg/mL in 1% FCS/PBS overnight at 4 °C. Nuclei were stained with 1 µg/mL DAPI (ROTH, Karlsruhe, Germany) in 1% FCS/PBS followed by three washing-steps with 1% FCS/PBS. Finally, the slides were mounted with Fluoromount-G (eBioscience, San Diego, CA, USA) and examined with a Leica DM2500 microscope (Leica, Wetzlar, Germany). Four to five random pictures at 100× magnification from each of 4 ear sheets per mouse were evaluated to determine the number of DETC per mm^2^. DETC were counted manually with the plugin “Cell Counter” in the Image J Software. Representative images were cropped from larger images and adjusted for brightness and contrast to improve clarity.

### 4.3. Isolation of Epidermal Cells

Epidermal cells were isolated from the back and belly skin of mice. Mice were sacrificed and their skin was dissected, freed from fat and incubated with dermal-side down on 0.75% trypsin in PBS (PAN-Biotech) for 1.5 h at 37 °C. The epidermis was gently peeled off from the dermis using forceps and further incubated in 0.05% trypsin/0.05% EDTA in PBS (PAN-Biotech) for 1 h at 37 °C followed by rigorous pipetting. The cell suspension was diluted with medium (DMEM, 10% FCS, 1% penicillin/streptomycin; PAN-Biotech) to stop the trypsin reaction, filtered through a 70 µm strainer (BD Biosciences) and subsequently stained for flow-cytometry. When indicated, epidermal cells were incubated overnight at 37 °C in culture medium (RPMI, 10% FBS, 1% Penicillin/Streptomycin; PAN-Biotech) for re-expression of the antibody-binding site on the Vγ3-TCR after trypsinization. Otherwise, DETC were identified by γδTCR-expression. Centrifugation of cell suspensions was performed at 200 × *g* for 5 min at 4 °C. 

### 4.4. Flow Cytometry

Before staining, Fc-receptors were blocked with 2.5 µg/mL anti-CD16/32 (clone 93; #101321 BioLegend, San Diego, CA, USA). Antibodies used for surface staining were anti-γδTCR-APC (clone GL3; #118116 BioLegend), anti-γδTCR-BV711 (clone GL3; #563994 BD Biosciences), anti-Vγ3TCR-FITC (clone 536; #553229 BD Biosciences), anti-IL10R-PE (clone 1B1.3a; #112706 BioLegend), and isotype-control rat IgG1κ-PE (clone RTK2071; #400408 BioLegend). Cells were stained for 20 min at 4 °C in FACS-buffer (2% FCS/PBS with 2 mM EDTA (Carl Roth, Karlsruhe, Germany)). Dead cells were identified by staining with 2 µg/mL DAPI (Carl Roth). DETC were analyzed and sorted on a BD Aria III™ using the BD DIVA™ 8.0 software. For sorting and analysis, we used sequential gating in two-dimensional plots. Forward-scatter and DAPI-staining were used to exclude doublets and dead cells according to flow-cytometry guidelines [76]. The purity of sorted cells was between 90%–98%. 

For intracellular staining, dead cells were stained before fixation with fixable viability dye-eFluor506 (eBioscience) according to the manufacturer´s instructions. Cells were subsequently fixed with 2% formalin/PBS for 30 min at 4 °C and permeabilized with 1 µg/mL saponin (Sigma-Aldrich) in FACS-buffer. F-actin was stained with 1 U/mL phalloidin-AlexaFluor594 (Invitrogen, Carlsbad, CA, USA) in 1 µg/mL saponin (Sigma-Aldrich) in FACS-buffer for 30 min at 4 °C. IFNγ and GM-CSF was stained with anti-IFNγ-APC (clone XMG1.2; #505810 BioLegend) and anti-GM-CSF-PE (MP1-22E9; #505406 BioLegend) in 1 µg/mL saponin (Sigma-Aldrich) in FACS-buffer for 20 min at 4 °C. Intracellular calcium was stained by incubation of unfixed cells with 4 µg/mL Fluo3AM (Invitrogen) in Hank’s balanced salt solution (Sigma-Aldrich) for 30 min at 37 °C. Acquired data were exported from the BD DIVA^TM^ 8.0 software as FCS 3.0 files and further analyzed in FlowJo^TM^ Software Version 10.6.1 (BD and Company, Ashland, OR, USA) 

### 4.5. Microarray Analysis

For animal breeding reasons *Ahr ^+/-^* mice littermates were used as controls for *Ahr ^-/-^* mice. We showed before that DETC in *Ahr ^+/-^* are not different from those in *Ahr ^+/+^* mice [4]. Four *Ahr ^-/-^* and three *Ahr ^+/-^* samples were created, each consisting of processed RNA pooled from DETC sorted from 2–3 mice 2 weeks of age. RNA from sorted DETC was extracted from frozen cell pellets using the NucleoSpin RNA XS kit (Macherey Nagel, Düren, Germany) according to the manufacturer’s protocol. All RNA samples were analyzed by photometric Nanodrop (ThermoFisher Scientific, Waltham, MA, USA) measurement and quantified by fluorometric Qubit RNA HS Assays (Life Technologies, Waltham, MA, USA). RNA samples were checked for RNA integrity using the RNA 6000 Pico Chip and the 2100 Bioanalyzer system (Agilent, Santa Clara, CA, USA). Only samples with high-quality RNA were included in this study. Synthesis of cDNA and subsequent biotin labeling of fragmented cDNA was performed according to the manufacturer’s protocol (GeneChip^®^ Pico Reagent Kit 703,308 Rev. 4, ThermoFisher scientific). Briefly, 10 ng of total RNA was converted to cDNA, amplified to complementary RNA (cRNA) followed by in vitro amplification and conversion to cDNA. After fragmentation cDNA was biotin-labeled and hybridized to Applied Biosystems^TM^ Clariom^TM^ S Mouse Gene Expression Microarrays for 16 h at 45 °C, stained by streptavidin-PE conjugate, and scanned as described in the manufacturer’s protocol. Microarray data analysis was performed using the software R [77] (3.5.2); quality control of hybridization data was performed with ArrayQualityMetrix (3.38.0) [78], a Bioconductor [79] package under R. The hybridization intensity values were normalized using the Robust Multichip Average algorithm (RMA) using the oligo (1.46.0) [80] package in R, which allows background subtraction, quantile normalization, and summarization (via median-polish). The differentially regulated genes were extracted using limma (3.38.3) package [81,82,83], then annotated using mogene20 package (chip mogene20sttransriptcluster) [84] False Discovery Rate (FDR) was addressed using the Benjamini and Hochberg’s method [85] and FDR has been used to measure the statistical significance, *p*-value < or = 0.05. 

To study the biological function of the differentially regulated genes DAVID Functional Annotation Bioinformatics Microarray Analysis Tools (http://david.abcc.ncifcrf.gov/) was used and the results were plotted in R using ggplot2 (2.3.1.1) package [86]. 

The normalized expression matrix was used to evaluate the gene set enrichment analysis (GSEA), which was performed using GSEA software [32,87], with the following settings: “metric to ranking genes” set to “*t*-test”, “Min size” set to 5, Gene sets database: c5.all.v6.2.symbols.gmt, Chip platform: Clariom_S_Mouse.r1.chip; all the remaining options were set to default values.

For the visualization of the GSEA-results, the EnrichmentMap-Plugin [88] in Cytoscape [89] was used according to the protocol of Reimand et al. 2019 [30].

### 4.6. Quantitative Realtime PCR (qPCR)

RNA from sorted cells was extracted from frozen cell pellets using the NucleoSpin RNA XS kit (Macherey Nagel) according to the manufacturer’s protocol. cDNA was prepared with random hexamer primers (Carl Roth), dithiothreitol (Sigma-Aldrich), RiboLock (ThermoFisher Scientific) and M-MLV reverse transcriptase, (Promega, Madison, WI, USA). qPCR was performed in a Qiagen rotor gene using Sybr-green master mix (Biorad, Hercules, CA, USA). All qPCRs were controlled for technical quality by excluding measurements, in which the technical replicates varied with SD > 0.5. CT-Values of genes of interest (GOI) were calibrated to the housekeeper gene *Rps6*, by calculating the ratio 2^(CT*Rps6* – CTGOI)^. Genes with CTs over 30 were assumed to be not detected. The limit of detection (lod) of most qPCRs was between the ratios 0.0005 and 0.0025 and was set to 0.0015 in the graphs, if not stated otherwise, to provide a reference for the comparison of expression levels between genes. Primer sequences are shown in Supplementary Materials.

### 4.7. Statistics

Data were tested for normal distribution by a Shapiro–Wilk-test and further analyzed by stated nonparametric Mann–Whitney or parametric *t*-tests using the software GraphPad Prism Version 8.3.0 (538) (GraphPad Software, LLC, San Diego, CA, USA).

## Figures and Tables

**Table 1:** Xenobiotic response elements (XRE) sequences in leading-edge genes of negatively associated pathways in DETC from *Ahr ^-/-^* mice.

Present in Pathways ^a^	Gene	FC ^b^	*p*-value ^c^	adjusted. *p*-value ^d^	XREs ^e^
10	***Arhgap6***	−1.716	0.000	0.008	2
7	*Rhod*	−0.998	0.006	0.149	5
6	***Ptpro***	−0.698	0.000	0.024	4
6	*Phldb2*	−0.459	0.029	0.332	7
5	***Fermt2***	−4.005	0.000	0.000	10
5	***Sparc***	−3.751	0.000	0.000	4
4	***Vcl***	−1.074	0.001	0.036	1
4	*Smad3*	−0.406	0.004	0.131	14
4	*Pdgfa*	−0.681	0.006	0.157	12
4	*Fbln1*	−0.376	0.028	0.330	10
3	***Nid2***	−3.194	0.000	0.000	5
3	***Palld***	−2.729	0.000	0.011	4
3	*Prkce*	−0.580	0.002	0.072	5
3	*Cd2ap*	−0.593	0.005	0.137	5
3	*Fmn1*	−0.291	0.041	0.387	4
3	*Aif1*	−0.286	0.041	0.387	6
2	***Rapgef3***	−1.133	0.000	0.005	4
2	***Kit***	−1.220	0.000	0.006	11
2	***Ahsg***	−0.750	0.000	0.014	4
2	***Cspg4***	−1.559	0.001	0.049	6
2	*Bbs2*	−0.704	0.001	0.059	5
2	*Numbl*	−0.451	0.005	0.133	9
2	*Kifc3*	−0.500	0.005	0.145	4
2	*Gcnt2*	−0.428	0.013	0.231	2
2	*Megf9*	−0.346	0.015	0.246	2
2	*Rab13*	−0.308	0.017	0.269	4
2	*Shank3*	−0.287	0.021	0.295	4
2	*Fgd6*	−0.638	0.026	0.319	5
2	*Aplp1*	−0.351	0.036	0.365	4
2	*Wdr35*	−0.390	0.039	0.377	7
2	*Stx2*	−0.492	0.042	0.393	5
1	***Podn***	−2.918	0.000	0.000	6
1	***Avil***	−1.803	0.000	0.000	6
1	***Kcng3***	−2.935	0.000	0.000	9
1	***Col27a1***	−2.178	0.000	0.001	10
1	***Rab38***	−1.393	0.000	0.001	1
1	***Abi3***	−1.422	0.000	0.002	4
1	***Mtss1***	−2.123	0.000	0.002	11
1	***Lrp5***	−1.109	0.000	0.003	8
1	***Nedd4l***	−0.809	0.000	0.007	8
1	***Il6st***	−1.866	0.000	0.008	2
1	***Islr***	−2.173	0.000	0.009	4
1	***Gng12***	−0.721	0.000	0.014	10
1	***Il12rb1***	−0.766	0.001	0.050	4
1	*Nisch*	−0.523	0.004	0.114	3
1	*Snca*	−0.382	0.004	0.128	6
1	*Ptpn13*	−0.575	0.005	0.145	3
1	*Stx3*	−0.427	0.008	0.184	3
1	*Tfrc*	−0.475	0.008	0.185	11
1	*Lrp6*	−0.508	0.009	0.188	4
1	*Pdgfrb*	−0.377	0.009	0.196	8
1	*Bcl11b*	−0.444	0.012	0.223	13
1	*Pard6b*	−0.561	0.013	0.228	7
1	*Cited1*	−0.458	0.015	0.252	13
1	*Cntnap4*	−0.308	0.019	0.279	8
1	*Arap1*	−0.395	0.019	0.281	7
1	*Vil1*	−0.365	0.019	0.283	7
1	*Stxbp1*	−0.374	0.021	0.291	7
1	*Kcna6*	−0.309	0.022	0.298	7
1	*Kcns2*	−0.294	0.023	0.303	6
1	*Hcn1*	−0.339	0.024	0.309	3
1	*Cxadr*	−1.192	0.027	0.322	9
1	*Sgk3*	−0.849	0.028	0.330	6
1	*Ptprm*	−0.418	0.033	0.353	5
1	*Prkcz*	−0.335	0.033	0.354	11
1	*Bsn*	−0.315	0.047	0.409	6

^a^ Number of negatively-associated pathways in which the respective genes were identified as a leading-edge gene. ^b^ FC = fold change. ^c^ *p*-value: nominal *p*-value without considering multiple testing (raw *p*-value corresponding to the t-statistics). ^d^ adjusted *p*-value corrected for multiple testing using the false discovery rate (FDR) method.^e^: Number of putative xenobiotic responsive elements (XREs) in the promotor of the respective gene according to Sun et al. 2004 [47]. Significantly changed genes (adjusted *p*-values) are in ***bold***.

## Supplementary Materials

Supplementary Materials can be found at https://www.mdpi.com/1422-0067/21/6/2249/s1.

## Abbreviations

AHR	Aryl hydrocarbon receptor
DETC	Dendritic epidermal T cells
AHRR	Aryl hydrocarbon receptor repressor
GSEA	Gene set enrichment analysis
lod	Limit of detection
FDR	False discovery rate
