# Supplementary material for: AHR Signaling Dampens Inflammatory Signature in Neonatal Skin γδ T Cells
Source: Int J Mol Sci. 2020 Mar 24;21(6):2249. doi: 10.3390/ijms21062249 (PMC7139545; doi:10.3390/ijms21062249)
Supplement: Supplementary file 1 [file ijms-21-02249-s001.pdf]

Article

AHR signaling dampens inflammatory signature in neonatal skin γδ T cells.

Katja Merches [1], Alfonso Schiavi [1,4], Heike Weighardt [2], Swantje Steinwachs [1], Nadine Teichweyde [1], Irmgard Förster [2], Katrin Hochrath [1], Beatrix Schumak [3], Natascia Ventura [1,4], Patrick Petzsch [5], Karl Köhrer [5] and Charlotte Esser [1,*]

1 IUF – Leibniz Research Institute for Environmental Medicine, Auf'm Hennekamp 50, D-40225 Düsseldorf, GERMANY
2 Immunology and Environment, Life & Medical Sciences (LIMES) Institute, University of Bonn, Carl-Troll-Str. 31, D-53115 Bonn, GERMANY;
3 Institute of Medical Microbiology, Immunology and Parasitology, University of Bonn, Venusberg Campus 1, D-53127 Bonn, GERMANY
4 Institute of Clinical Chemistry and Laboratory Diagnostic, University of Düsseldorf, Universitätsstrasse 1, D-40225 Düsseldorf, GERMANY
4 BMFZ – Genomics & Transcriptomics Laboratory, University of Düsseldorf, Universitätsstrasse 1, D-40225 Düsseldorf, GERMANY

* Correspondence: Charlotte.Esser@iuf-duesseldorf.de; Tel.: +49-211-3389-253

Supplementary Data

Supplementary Materials

Supplemental Table 1

Primer sequences for quantitative real-time PCR

Rps6 (TACTGTGCCTCGTCGGTTG, antisense: TGAATCTTGGGTGCTTTGGT), *Ifnγ* (GGCCATCAGCAACAACATAA , antisense: ATCAGCAGCGACTCCTTTTC), *Pdl1* (AAGTCAATGCCCCATACCGC, antisense: CTCTTCCCACTCACGGGTTG), *Ccr5* (AGACATCCGTTCCCCCTACA, antisense: GCAGGGTGCTGACATACCAT), *Ccl1* (GCATGCTTACGGTCTCCAAT, antisense: AGGCGCAGCTTTCTCTACCT), *Ccr2* (CAGAAGAGGGCATTGGATTC, antisense: CAAGGCTCACCATCATCGTA), *Ccr1* (GTTGGGACCTTGAACCTTGA, antisense: TCCTTTGCTGAGGAACTGGT), *Kcnma1* (ATATCCGCCCAGACACTGAC, antisense: ATCGTTGGCTGCAATAAACC), *Camk2d* (TCCATCGGGACCTAAAGCCT, antisense: TGCCAGCAAAACCAAACCAT), *Fermt2* (CCCAGAGATCCCACAAAGAA, antisense: TATCCCAGGATTGCCTTCTG),

Arhgap6 (TTTGACCGTGGGGTTGATGT, antisense: GGCTCCAACAGGAGAGTGTT), *Rhod* (AACCTGTGCACCTCCAAATC, antisense: CACAACAATGATGGGCACTC), *Advillin* (GTGGAGGTGGATCAGCAGTT, antisense: GTTGGAGTTGAGCGAGGAAG), *Il10rα* (GCCCTCAAACAGTACGGAAA, antisense: AAGCGAGTCTCAGTGGTGGT), *Scinderin* (CAGGGTGCCTATGGTGACTT, antisense: CTTGCCACCCAAATAGTCGT), *GranzymeF* (TGACCCTTCTTCTGCCTCTC, antisense: TCATTGAGCTTCCAGTGCAG), *C-Kit* (ATCCCGACTTTGTCAGATGG, antisense: AAGGCCAACCAGGAAAAGTT), *Rpl39* (CAAAATCGCCCTATTCCTCA, antisense: TTCAAGACACGAAGAGTCTCCA), *Isg2*0 (CCATGACCTGAAGCACGACT, antisense: CCCGCCAGTTGTTCTGGAT), *Xrn2* (ACAGCAACTGTATTACCCCAGG, antisense: GGCTGGGCTCTTTGTCTTCT), *Kif2c* (GCCGACACATCTAGTGCTGA, antisense: GAGTTCTCCCCAATGAACGA), *Smc5* (AATCCTTCGGTGGTTGAGGAG, antisense: CCACCTTTCTTTCACCTGCG), *Nek2* (CCTGGACAGCAAACACAATG, antisense: CAAGGACCAGATGTCCGACT), *Cyp1a1* (ATATCCCCAAGGGGTGCTGT, antisense: TTCAGGCCGGAACTCGTTTG)

Supplementary Figure 1

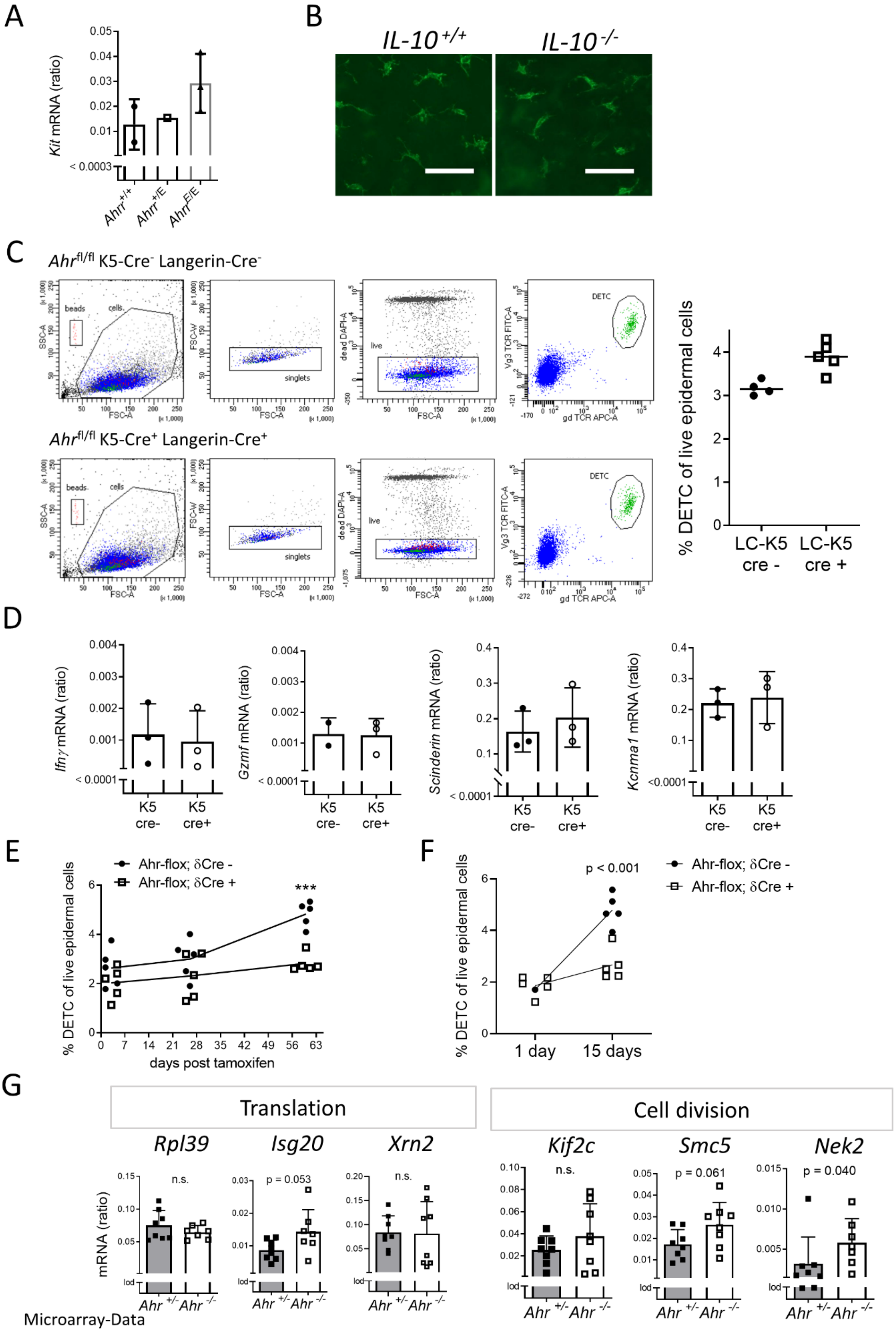

Microarray-Data

	Rpl39	Isg20	Xrn2	Kif2c	Smc5	Nek2
Fold-Change:	0.717	1.141	0.956	0.934	1.079	0.730
Adj. P-Value:	0.165	0.012	0.083	0.325	0.006	0.379

Supplementary Figure 1

(A) RNA from sorted DETC from 6 – 32 weeks old *Ahrr* $^{+/+}$, *Ahrr* $^{+/E}$ or *Ahrr* $^{E/E}$ mice was isolated and expression of *Kit* was measured by qPCR. Shown are ratios referred to the housekeeper gene *Rps6* (n = 1-3 mice) (B) Representative pictures of epidermal ear sheets from 9-20 weeks old *Il-10* $^{+/+}$ or *Il-10* $^{-/-}$ mice stained with anti-Vγ3 (scale bar = 50 µm; n = 3 mice) (C) Epidermal cells from female, 9 weeks old, *Ahr* $^{fl/fl}$ Cre- or *Ahr* $^{fl/fl}$ K5-Cre+Langerin-Cre+ mice (LC-K5-Cre+) were stained for DETC (Vγ3+, γδTCR+) and were analyzed by flow cytometry for the frequency of DETC (n = 4-5 mice). (D) Epidermal cells from male and female, 13-16 weeks old, *Ahr* $^{fl/fl}$ Cre- or *Ahr* $^{fl/fl}$ K5-Cre+ mice were stained for DETC (Vγ3+, γδTCR+) and DETC were FACS-sorted to over 98 % purity and their RNA was isolated. Expression of interferon-γ (*Ifnγ*), granzyme F (*Gzmf*), scinderin (*Scin*) and *Kcnma1* was measured by qPCR; shown are ratios referred to the housekeeper gene *Rps6* (n = 3 mice) (E) 8 week old Ahr-flox; δCre+ and δCre- mice were treated with tamoxifen as described in the methods section. Epidermal cells were isolated 3, 26 and 60 days after the last tamoxifen-injection and stained for DETC by anti-γδTCR and anti-Vγ3 antibodies. The percentage of DETC from live cells is displayed (2-way-ANOVA with Sidak's test for multiple comparisons, *** $p < 0.001$; n = 5 mice) (F) Newborn mice (1 day old) were treated with tamoxifen as described in the method section. Epidermal cells were isolated 1 day and 15 days after the last tamoxifen-treatment and stained for DETC by anti-γδTCR and anti-Vγ3 antibodies. The percentage of DETC from live cells is displayed (t-test for 15 days timepoint; n = 1-5 mice) (G) RNA of DETC, FACS-sorted from mice two weeks of age, was isolated. Expression of indicated genes was measured by qPCR; shown are ratios to the housekeeper gene *Rps6* (n = 8; Mann-Whitney-test for *Nek2*, t-tests for remaining genes)

Supplementary Figure 2

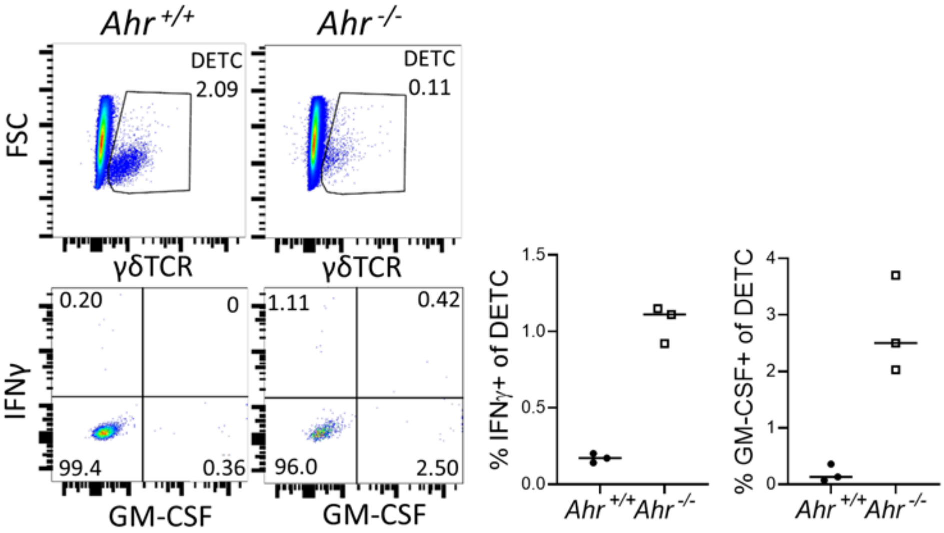

Supplementary Figure 2

Epidermal cells were isolated from 8 weeks old *Ahr* $^{+/+}$ and *Ahr* $^{-/-}$ mice and stained for DETC by anti-γδTCR-BV711 followed by fixation and intracellular staining for IFNγ by anti-IFNγ-APC and for GM-CSF by anti-GM-CSF-PE. One representative plot for DETC-gating from live cells and IFNγ/GM-CSF proportion among DETC is shown per genotype. The plots were not standardized to a certain cell number. The percentage of IFNγ+ or GM-CSF+ DETC of all DETC is displayed as graph (n = 3 mice)
